# Racial and Ethnic Differences in Retirement Satisfaction among Older US Adults

**DOI:** 10.1093/geroni/igab046.3287

**Published:** 2021-12-17

**Authors:** Samuel Van Vleet, Sara McLaughlin

**Affiliations:** Miami University, Oxford, Ohio, United States

## Abstract

Along with population aging, the diversity of the older US population is increasing. Research suggests that racial and ethnic minorities experience disadvantages over the life course that can negatively impact later life. Despite this, little research has examined racial and ethnic differences in satisfaction with retirement. Using data from the 2016 wave of the Health and Retirement Study, we compared retirement satisfaction among Black (n = 1,068), Hispanic (n = 674), Other (n=161), and White (n = 4,833) older adults (age 65+). Retirement satisfaction was measured with the following item: “All in all, would you say that your retirement has turned out to be very satisfying, moderately satisfying, or not at all satisfying?” Responses were categorized as very satisfied vs. all others. Approximately 43% of Black, 35% of Hispanic, 39% of Other, and 56% of White Americans reported being very satisfied with retirement (χ2(2.4)=58.9; p < .0001). In multivariate logistic models controlling for age, educational level, gender, household income, marital status, and functional limitations, the odds of being very satisfied with retirement were 32% lower for Hispanic (OR=0.68; 95% CI= 0.55, 0.85) and 37% lower for Other Americans (OR=0.63; 95% CI= 0.43,0.92) relative to their White counterparts. No significant difference was evident for Black and White Americans in adjusted analysis (OR=0.96; 95% CI=0.76,1.20). Our findings indicate that inequalities in the retirement experience exist by race and ethnicity in the United States. More research is needed to understand the factors responsible for lower retirement satisfaction among Hispanic and Other Americans.

